# In vitro antioxidant and cytotoxicity activities of selected indigenous South African medicinal plants

**DOI:** 10.4314/ahs.v22i1.48

**Published:** 2022-03

**Authors:** Yonela Vakele, Frederick Odun-Ayo, Lalini Reddy

**Affiliations:** 1 Department of Horticultural Science, Cape Peninsula University of Technology, Cape Town, South Africa; 2 Department of Biotechnology and Consumer Sciences, Cape Peninsula University of Technology, Cape Town, South Africa

**Keywords:** In vitro antioxidant, cytotoxicity activities, South African medicinal plants, *C. comosum, K. uvaria, T. violacea.*

## Abstract

**Background:**

Medicinal plants are regarded as a large source of phytochemicals that may have anticancer properties. This could lead to the development of innovative drugs or alternative therapy against cancer.

**Objective:**

This study was designed to determine the antioxidant and cytotoxicity effect of 5 selected indigenous South African medicinal plants namely; *Bulbine frutescens, Bulbine natalensis, Chlorophytum comosum, Kniphofia uvaria*, and *Tulbaghia violacea*.

**Method:**

Phytochemical extracts namely; methanol, 50%, 100% ethanol, and water extracts were prepared from the root and shoot of the plants. The antioxidant effect of methanol extracts of the plant materials was performed using a DPPH assay. A preliminary cytotoxicity screening of the phytochemical extracts in the human colon (Caco-2), cervical (HeLa), and hepatocellular (HepG2) cell lines were determined followed by the half-maximal inhibitory concentration (IC50) using MTT assay.

**Result:**

The methanol root extract of *B. natalensis* and *B. frutescens* (33.20% and 26.33% respectively) and shoot extract of *K. uvaria* (17.10%) showed the highest antioxidant. Out of the 5 plants, only 100% ethanol extract of *C. comosum, K. uvaria*, and *T. violacea* caused more than 80% cytotoxicity in HepG2 and Caco-2 cell lines. The shoot of *B. frutescens* (10.43 µg/ml), *K. uvaria* (23.0 µg/ml), and root of *C. comosum* (23.77 µg/ml) were the most active with the highest cytotoxicity.

**Conclusion:**

*C. comosum, K. uvaria*, and *T. violacea* possess significant cytotoxicity that is promising in developing alternative drugs against colon and liver cancers. Our results provided new pieces of evidence for antioxidant and cytotoxic activities of these plants which could be useful for developing new anticancer therapies.

## Introduction

Cancer is a life-threatening disease that severely affects the human population^1^. It is characterized by the uncontrollable or unstopped tumours of malignant cells in the human body that has the potential to be metastatic^2^. The current treatment of cancer is by chemotherapy, radiotherapy, and chemically derived drugs which give side effects causing a lot of strain and health damage on the patient. Hence, the need to focus on developing alternative and effective treatments against cancer. The use of natural products as anticancer agents was recognized by U.S. National Cancer Institute (NCI) in the 1950s^3^. Some the medicinal plants have been revealed to have both chemopreventive and/or therapeutic effects on breast cancer^4^ and skin cacer, and most clinically useful ati-cancer agents are sourced from plant-derived compounds^3^. Some medicinal plants have been revealed to have both chemopreventive and/or therapeutic effects on breast cancer^4^ and skin cancer, and most clinically useful anti-cancer agents are sourced from plant-derived compounds^3^.

Southern Africa has over 30000 species of higher plants with most of them possessing medicinal properties which are promising for further development and commercialization^5^. Table 1 shows the biological activities of some selected South African medicinal plants and their indigenous use for the treatment of ailments.

**Table 1:** Indigenous treatments and biological activities of selected South African medicinal plants

Scientific name (Family)	Distribution and Propagation	Part(s) used	Indigenous treatment usage	Study models	Reported biological properties
*Bulbine frutescens* (Asphodelaceae)	It is widely distributed in the Northern Cape, Western and Eastern Cape of South Africa. Propagates in spring by seeds, cuttings, or by division of clumps^18^	Bulbs Leaves	Treatment of diarrhoea^14^, skin wounds and burns^19^.	Human hepatocellular HepG2, human breast (MDA-MB- 231 and T47D), and embryonic kidney 293 (HEK293) cancer cells.	Antibacterial^14^, Apoptosis^16^,^17^
*Bulbine natalensis* (Asphodelaceae)	It is widely distributed in the Eastern and Northern parts of South Africa. Propagated from seeds during warmer seasons^10^.	Roots Leaves Bulbs	Stop bleeding, wounds, cracked lips, cuts, grazes, itches, mosquito bites, rashes, ringworm, sores, and herpes^5^.	Human hepatocellular HepG2 cell line.	Antibacterial^14^, Apoptosis^16^,^17^, Anti-diabetic^20^
*Chlorophytum* *comosum* (Agavaceae)	It is widely distributed from Swellendam (Western Cape) to the Soutpansberg (Limpopo) province. It can be propagated vegetatively by the division of the plantlets on the inflorescences. Various cultivars are *Chlorophytum* *comosum ‘Variegatum’,* *‘vittatum*, and *‘Mboyeti’*^21^	Roots Leaves	Bronchitis, asthma, fractures, and burns^22^.	Lung (A549) and breast (MCF-7), human cervical adenocarcinoma (HeLa), promyelocytic leukemia (HL-60), human T cell leukemia (CCRF-HSB-2), human monocyte tumor (U937) cancer cells.	Antioxidant and Antiproliferative^12^ Apoptosis^23^
*Kniphofia uvaria* (Asphodelaceae)	It occurs naturally in all the nine provinces of South Africa. KwaZulu- Natal possesses the highest number of *Kniphofia* species compared to the other provinces It can be propagated by seeds or division^25^	Roots Analogue derivatives	Asthma^26^; abdominal cramps and wound healing^28^	Mouse melanoma (B16), mouse monocyte/macropha ge tumour (RAW 264.7), human acute monocytic (THP-1), and promonocytic leukaemic (U937) cell lines	Antimalarial^27^, Anti-inflammation, Antioxidant^29^,^30^, Apoptosis^15^
*Tulbaghia* *violacea* (Alliaceae)	It is distributed from the Eastern Cape, KwaZulu- Natal and Limpopo, to as far north as Zimbabwe. This plant may be propagated either by seeds in spring or by dividing larger	Roots Leaves Bulbs	Type-1 diabetes, fever, colds, paralysis, hypertension, asthma, tuberculosis, inflammation^32^.	Colon adenocarcinoma (HT-29), cervical carcinoma (HeLa), and breast carcinoma (MCF-7) cell lines	Apoptosis and antiproliferative^13^:

These South African indigenous plant species come from different families such as Asphodelaceae, Alliaceae, Hypo xidaceae, Agavaceae and Asteraceae. For centuries, these indigenous medicinal plants have been adopted for primary health care and treatments^6^. Medicinal plants or their secondary metabolites have been known for directly or indirectly playing a vital role in the health of people to fight diseases^7^.

Secondary metabolites that are derived from plants are being investigated for the development of new clinical drugs to treat cancer^1^ and their ability to inhibit growth or initiate apoptotic cancerous cells. Furthermore, these compounds have properties that inhibit the proliferation of cancer cells and induce tapoptotic cell death. Some plants have been reported to be sources of natural antioxidants that can protect against oxidative stress and play a vital role in the chemoprevention of cancer^8^.

Medicinal plants commonly known as herbs, pharmacologically active plants remain the dominant form of medicine in most countries.

These plants are regarded as a large source of therapeutic phytochemicals (phenolic and flavonoids) that may lead to the development of innovative drugs against cancer^9^.

In this present study, 5 indigenous South African medicinal plants, namely; *Bulbine frutescens, Bulbine na talensis, Chlorophytum comosum, Kniphofia uvaria*, and *Tulbaghia violacea* were selected and tested for antioxidant and cytotoxicity activities. These selected medicinal plants have been used as traditional medicines for decades against diseases^5^,^10^. Because most communities in South Africa rely on traditional healers and medicines for primary healthcare, it is critical to validate herbal remedies while also preserving indigenous medicinal plants for the benefit of these communities. Although very few available studies have shown the anticancer potential of these plants^1^,^3^,^12^–^17^, more research needs to be performed to understand the cytotoxicity properties. To the best of our knowledge, only a few reports about their cytotoxicity activities have been published so far. There is little or no report of *Bulbine frutescens, Bulbine natalensis*, and *Chlorophytum comosum* cytotoxicity activities in human colon (Caco-2) and cervical (HeLa) cancer cell lines. Similarly, more information is needed regarding cytotoxicity activities of *Kniphofia uvaria* and *Tulbaghia violacea,* particularly, against hepatocellular (HepG2) cancer cell lines. Hopefully, our results may provide new information for anticancer drug development from these plants, particularly, against the aforementioned cancer cell line.

## Materials and Methods

### Plant materials

*Bulbine frutescens, Bulbine natalensis, Chlorophytum comosum*, *Kniphofia uvaria*, and *Tulbaghia violacea* were obtained from Shadowlands Wholesale Nursery at Kuilsriver, Cape Town, South Africa. Plants were transplanted in potting soil and kept in an environmentally controlled greenhouse for steady and healthy growth. After 12 weeks, the shoot and root of the plants were removed individually from all selected plants. The plant materials were washed thoroughly with distilled water to ensure the removal of all the dirt, air-dried at 28°C for 4–8 days and then oven-dried at 48°C to remove residual moisture content.

### Preparation of plant extracts

The dried plant materials were blended to a fine powder and stored at room temperature. Extracts were prepared as per methods described by Sousa et al.^33^. For each root and shoot methanol, ethanol, and water extracts, 90 g in 100 ml of methanol; 25 g in 100 ml of a prepared 50%; 100% ethanol, and 25 g in 200 ml of sterile distilled water were prepared. These were placed in a shaker at 156 rpm in an incubator of 37°C for 48 h. Plant extracts were filtered through a 40um filter disk (Whatman No 1, UK) and centrifuged at the speed of 4000 rpm for 20 minutes. The methanol and ethanol filtered extracts were concentrated in a rotatory evaporator (Buchi Rotavapor R-200, United States) and freeze-dried for 24 – 48 hrs. One (1) mg of the extract was dissolved in 1 ml of 10 mM plant tissue culture grade Dimethyl Sulfoxide (DMSO; Sigma-Aldrich, South Africa) to yield a concentration of 1000 µg/ml and stored at -20°C. All extracts were mixed thoroughly for homogeneity before the assay.

### Preparation of cell culture

The human Caco-2, HeLa, and HepG2 cancer cell lines were obtained from Biolabels Node, University of Western Cape, Cape Town, South Africa. The cell cultures were prepared as previously described by Matsushita et al.^23^ The cells were cultured with Dulbecco's Modified Eagle Medium (DMEM) supplemented with 10% foetal bovine serum (FBS), 1% pen-strep cocktail of 100 µg/ml penicillin and 100 µg/ml streptomycin. The cells were grown at 37°C in a 5% CO_2_ humidified incubator (Labotech, South Africa). The cells were seeded at 1 x 10^5^ cells/ml density in a 96-well plate and incubated at 37 °C for 24 hrs. The viability of cells was evaluated by direct observation under the EVOS XL core imaging system (Invitrogen, SA).

All cell lines were maintained in a humidified incubator at 37°C in an atmosphere of 5% CO_2_.

### Determination of antioxidant using 2, 2-Diphenyl-1-Picrylhydrazyl (DPPH) Assay

The antioxidant activity of the selected plants was measured in terms of radical scavenging ability using the stable radical DPPH as previously described by Kedare and Singh^34^ with slight modification. The DPPH is a stable radical in solution and appears purple colour absorbing at 515 nm in methanol. The hydrogen atom donating ability of the plant extracts changes the colour to yellow in the presence of antioxidants with a concomitant decrease in absorbance which was monitored by spectrophotometry^35^. For the assay, 125 µl of each plant material (root and shoot) methanol extracts were added to 150 µl of the DPPH solution in each of the 96-well plates. For the standards, 0.0125 g of Trolox (water-soluble antioxidant analogue) was diluted in 50 ml of 75% ethanol and was thoroughly mixed with a vortex until well dissolved. To prevent oxidation, plates were covered with aluminum foil and kept in the dark at room temperature for 1 hour. All samples were done in triplicates. The absorbance of the mixture was measured at 517 nm from the multiscan spectrum microplate reader. The percentage inhibition of the DPPH radical was calculated using the following formula:

$$\text{DPPH inhibition (\%) = 100 }\left[\frac{\text{Absorbance of a control sample - Absorbance of a tested sample}}{\text{Absorbance of a control sample}}\right]$$

### Determination of cytotoxicity activity using 3, 4, 5-dimethylthiazol-2-yl-2, 5-diphenyltetrazolium bromide (MTT) Assay

The mitochondrial activity of cells is measured using a spectrophotometer through the reduction of a yellow-coloured MTT salt to purple formazan crystals by the enzyme succinate tetrazolium reductase or succinate dehydrogenase, present only in metabolically active cells. The greater the purple colour of the reaction, the less the extent to which a particular compound (crude fraction) induces cell death in the specific reaction. The cytotoxicity of the plant extracts in the cell lines was measured by two rounds of screening procedures in succession using MTT assay as previously described by Liang et al.^36^. The stock solution of the compound was filtered through a 0.22 µm Millipore syringe filter to ensure sterility. A freshly prepared stock compound in 100% DMSO was made up to 100 µg/ml using 5% DMEM. A preliminary screening procedure was performed by assaying the cytotoxicity of the plant phytochemical extracts on the Caco-2, HeLa, and HepG2 cell lines seeded in separate 96 well microtiter plate (100 µl/well) and treated with 100 µg/ml concentration of the 50%, 100% ethanol extracts and aqueous extract of each plant materials for 24 hrs. After treatment, 10 µl of 5 mg/ml MTT (Sigma, USA) was added to each of the respective cell wells and control wells (containing 10% DMSO and cell lines) in triplicates and were incubated at 37°C for 3 hrs. The insoluble formazan crystals were solubilized by adding 100µl DMSO. The reduction of MTT (absorbance) was read at 570 nm using a microplate reader (Polar Star Omega). Cytotoxicity was calculated based on the following formula:

$$\text{Cytotoxicity (\%) = 100 - }\left[\frac{\text{Average absorbance of treated cells}}{\text{Average absorbance of control}}\right]100$$

### Determination of IC_50_ for active compounds

An extract showing more than 80% cell cytotoxicity on at least 1 human cancer cell line in the preliminary screening was put into the secondary screening to assay its dose-dependent cytotoxic effects on the human cancer cell line. The half-maximal cytotoxic concentration of the active compounds that caused 50% cell growth inhibition (IC50) was investigated was performed based on the method previously described by Liang et al. 36 on the human cancer cell line which shows the highest cytotoxicity. The cells were cultured as above and treated with increasing concentrationof plant extracts (0 – 100 µg/ml) for 24 h. Following the MTT assay, the IC50 was estimated using GraphPad Prism software version 5 (GraphPad Software, California, USA). The experiment was done in triplicate for each assay.

## Results

### Antioxidant activity by DPPH assay

**Figure 1:**
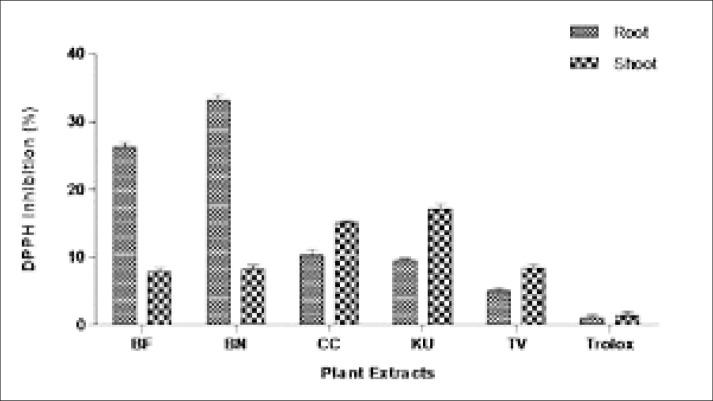
DPPH inhibition by roots and shoots in BF= *Bulbine frutescens*, BN= *Bulbine natalensis*, CC= *Chlorophytum comosum*, KU= *Kniphofia uvaria*, TV= *Tulbaghia violacea,* and Control: Trolox

The antioxidant of the root and shoot methanol extracts of *B. frutescens, B. natalensis, C. comosum, K. uvaria*, and *T. violacea* was determined through the percentage of DDPH inhibition by the plant extracts in the assay. The antioxidant activity through DPPH inhibiton assay of the 900 µg/ml methanol plant extracts is shown in Figure 1. In the root extract, *B. natalensis* (33.20%) and *B. frutescens* (26.33%) were found to exhibit the highest antioxidant in comparison to the root extract of other plants. Both *C. comosum* (10.33%) and *K. uvaria* (9.60%) showed a low level of antioxidant index, although it was significantly higher (p<0.05) when compared with *T. violacea* extracts (5.06%). In the shoot extract, the result showed that the free radicals were inhibited the most by *K. uvaria* (17.10%) and *C. comosum* (15.13%) compared to *T. violacea* (8.43%), *B. natalensis* (8.16%), and *B. frutescens* (7.86%).

### Cytotoxicity of the plant extracts using MTT assay

**Experiment I:** Selection of cytotoxicity above 80% cell growth inhibition.

A preliminary assay was performed to evaluate the cytotoxicity effect above 80% cell growth inhibition of 50%, 100% ethanol, and aqueous extracts of the roots and shoots of *B. frutescens, B. natalensis, C. comosum*, *K. uvaria* and *T. violacea* in Caco-2, HeLa and HepG2 cell lines using MTT assay. It was observed that only 100% ethanol extract of 3 plants (*C. comosum, K. uvaria*, and *T. violacea*) caused more than 80% cytotoxicity against HepG2 and Caco-2 cell lines as shown in Table 2. The highest cytotoxicity was noted in HepG2 treated with the 100% ethanol (root) extract of *C. comosum* (90.47%) and *T. violacea* (90.20%). It was only *C. comosum* (root) that inhibits growth (88.20%) of Caco-2 compared to other root plant extracts. For the100% ethanol (shoot) extracts, *K. uvaria* inhibits (85.43%) growth of Caco-2. In HepG2, T. violacea and *K. uvaria* caused high cytotoxic activity (85.43% and 84.43%) respectively, while that of *B. frutescens* (71.56%) and *B. natalensis* (72.87%) cytotoxicity was high but not above 80%. It was noted that none of 50% ethanol and aqueous extracts of the selected 5 plants showed above 50% cytotoxicity against the cell lines. Also, none of the plant material extracts resulted in a cytotoxic effect > 80% against the HeLa cell line. To further confirm the cytotoxicity of plant extracts showing more than 80% cytotoxicity in the cell lines, we assayed all the extracts against HepG2 which was most sensitive to the 100% ethanol plant extract to determine the dose-dependent half-maximal cytotoxic concentration and the most active plant extracts that caused 50% cell growth inhibition (IC50). The 100% ethanol extracts for all the plants showed a stronger cytotoxic effect against HepG2 cells except for *B. natalensis*. The 100% ethanol extracts for *B. frutescens* (shoot), *K. uvaria* (shoot) and *C. comosum* (root) were the most active with IC50 = 10.43 µg/ml, IC50 = 23.0 µg/ml and IC50 = 23.77 µg/ml respectively exhibiting the highest cytotoxicity as shown in Table 3. The *B. frutescens* (root) IC50 value was 32.72 µg/ml and it was the only 50% ethanol extract amongst all the plant extracts to exhibit cytotoxic effect with IC50 < 50 µg/ml. The shoot of *T. violacea* was shown to have significant cytotoxicity with IC50 < 50 µg/ml

**Table 2:** Cytotoxicity of the phytochemical extracts of the indigenous plants on Caco-2, HeLa, and HepG2 cells

Plant name (Botanical)	Extract	Cytotoxicity (%)					
		Caco-2		HeLa		HepG2	
		Root	Shoots	Root	Shoots	Roots	Shoots
*Bulbine frutescens*	WE	10.00	13.25	0.00	0.00	0.00	0.00
	50% EE	0.00	0.00	35.32	0.00	5.72	29.37
	100% EE	20.10	22.58	0.00	32.39	67.83	71.56
*Bulbine natalensis*	WE	0.00	13.25	23.16	0.00	23.16	40.31
	50% EE	0.00	0.00	0.00	0.00	26.34	17.97
	100% EE	8.52	44.50	0.00	50.24	33.73	72.87
*Chlorophytum comosum*	WE	37.31	20.54	10.10	0.00	13.5	0.00
	50% EE	23.20	24.58	5.85	26.90	22.41	13.78
	**100% EE**	**88.20**	43.43	45.23	0.00	**90.47**	22.31
*Kniphofia uvaria*	WE	15.60	17.66	5.50	15.89	40.50	4.65
	50% EE	0.00	0.00	0.00	0.00	46.61	24.85
	**100% EE**	33.61	**85.43**	0.00	75.30	77.23	**84.43**
*Tulbaghia violacea*	WE	0.00	0.00	0.00	0.00	0.00	32.34
	50% EE	10.10	0.00	0.00	0.00	13.54	11.12
	**100% EE**	77.23	72.89	34.20	21.50	**90.20**	**85.43**

WE: water extracts; EE: Ethanol extract; Extracts that caused more than 80% cytotoxicity on the cell lines assayed are in bold. Cytotoxicity (%) was presented athe mean of three replicates.

**Table 3:** IC_50_ values of the extracts against HepG2 cells

Plant name (Botanical)	Extract	IC_50_ (µg/ml) HepG2	
		Root	Shoots
*Bulbine frutescens*	WE	>100	>100
	**50% EE**	>100	34.00
	**100% EE**	**32.72**	**10.43**
*Bulbine natalensis*	WE	>100	74.23
	50% EE	66.65	100
	100% EE	>100	61.19
*Chlorophytum comosum*	WE	>100	>100
	50% EE	>100	>100
	**100% EE**	**23.77**	>100
*Kniphofia uvaria*	WE	100	>100
	50% EE	>100	>100
	**100% EE**	100	**23.00**
*Tulbaghia violacea*	WE	>100	>100
	50% EE	>100	>100
	**100% EE**	78.6	**45.61**

WE: water extracts; EE: Ethanol extract; Extracts that caused half-maximal inhibitory inhibition (IC_50_) on the HepG2 cell line assayed are in bold.

## Discussion

In this present study, we evaluated the antioxidant effect of the phytochemicals that scavenged free radicals in the DPPH assay. The root methanolic extract of *Bulbine frutescens* and *Bulbine natalensis* showed high antioxidant ativities compared to the extracts from the shoot of both plants and the root and shoot extracts of *T. violacea* with low antioxidants. This possibly means the root part of the *Bulbine* sp contains sensitive phytochemicals capable of scavenging free radicals. A previous study reported that *B. frutescens* contain secondary metabolites such as quinones and glycoside which are potential anticancer agent^37^. In our study, the shoot methanolic extract of *C. comosum* showed high antioxidant activity. In contrast, a previous study reported the root methanolic extract of *C. comosum* exhibited a strong and significant antioxidant activity^38^. 24reported less antioxidant response by *C. comosum* root extract in comparison to the ethanol and aqueous leave extracts. The concentration of the methanol solvents could affect the phenolic content which is a strong antioxidant agent in plant extracts^39^. Out of the 5 selected plants in this study, 100% ethanol extract of *B. frutescens* (root and shoot), *K. uvaria* (shoot), and *C. comosum* (root) were confirmed the most active plant extracts showing the great cytotoxic effect (> 80%) and the IC_50_ values were between 10.0 – 35.0 µg/ml in HepG2. However, we noticed that *T. violacea* recorded high cytotoxicity with IC_50_ = 45.61 µg/ml. 16reported that methanol extract of B. frutescens induced reactive oxygen species (ROS) by 7%n cancer cell lines when compared to the untreated cells. Although, cancer cells may develop defense mechanisms against antioxidants and anti-proliferative agents which counters the effect of elevated ROS. Excessive production of ROS and potential changes in mitochondrial membrane is associated with the initiation of cell death^40^. *B. frutescens* methanol and hexane extracts induced cell cycle arrest, ROS production, apoptosis, and mitochondria membrane potential disruption in breast cancer cells^16^. In our study, the shoot 100% ethanol extract of both the *Bulbine* sp induced potent cytotoxicity between 70 – 72% with B. frutescens IC50 value < 50 µg/ml compared to *B. natalensis* > 50 µg/ml. 17reported *B. natalensis* and *B. frutescens* induced a cytotoxic effect in the human laryngeal carcinoma cell line, however, the expression of caspase-3 which indicates apoptotic pathway was induced in shoot and leaf ethanol extracts except for the root of *B. frutescens*. Our study corroborates with previous studies which showed root extracts of *C. comosum* possess anti-proliferative activity against many human cancer cell lines. 24revealed saponins as major constituents in roots of *C. comosum. S*aponins isolated from roots of *C. comosum* exhibited cytotoxic and antitumour promoter activity through induced apoptosis in selected cancer cell lines^23^. To the best of our knowledge, for the first time, our study investigated the antioxidant activity of the phytochemical extracts from *K. uvaria*. The shoot extract of *K. uvaria* presented the highest antioxidant value compared to the other plants while the root extracts showed moderate antioxidant activity. *K. uvaria* is one of the most traditional medicinal herbs used for antimalaria and anti-inflammation but so far there is no research on the bioactivities related to its chemical components. We noticed there is little or no literature report on *K. uvaria*, however, *K. foliosa* through its derivative compounds, knipholone and knipholone anthrone demonstrated poor and potent antioxidant activity respectively^15^ and relatively little cytotoxic effect on human cell lines^30^. Previous phytochemistry studies have shown that roots of *K. foliosa* contain anthraquinones, flavonoids, and alkaloids which have anticancer activities^15^,^29^. In our study, the 100% ethanol extract of *T. violacea* induced a high cytotoxic effect in HepG2 and Caco-2 when compared to HeLa cell lines. 13 reported shoot methanol extract of *T. violacea* inhibited the growth of HeLa and HT-29 (colon cancer) cell lines. This was similar to our result which also showed a moderate degree of inhibition in both HeLa and Caco-2 cell lines. However, our study showed that the inhibition was high in the Caco-2 compared to HeLa as opposed 13 report showing high inhibition in HeLa in comparison to HT-2922. This could plausibly be the effect of the different solvent types used to extract the phytochemicals. Phytochemical compounds present in different parts of *T. violacea* may vary and these include tannins, terpenoids, flavonoids, saponins, proteins, steroids, cardiac glycosides, phenols and coumarins^41^

We noticed the aqueous extract has little or no cytotoxic effect on the selected cell lines. Previous studies showed that the aqueous extract from *T. violacea* exhibited apoptotic effect, although through a compound moiety-methyl-α-D-glucopyranoside which serves as a potential anticancer agent by interfering with hexokinase to inhibit the induction of ROS^42^. The high cytotoxic effect exhibited by the 100% ethanol extract of *T. violacea* in HepG2 compared to other plants or cell lines could suggest that cytotoxicity was induced by an apoptotic pathway that could be caspase-3 dependent^13^. This also suggests that the selectivity of *T. violacea* extracts and cell lines to text its cytotoxicity is an important factor to always consider for future studies

## Conclusion

The invitro antioxidant and cytotoxicity activities of B. frutescens, *B. natalensis, C. comosum, K. uvaria* and *T. violacea* against the selected human cancer cell lines implies the potential therapeutic source which can be utilized towards the development of new anticancer drugs. It is evident from this study that C. comosum, *K. uvaria*, and *T. violacea* possess significant cytotoxicity that is promising in developing alternative drugs against colon (Caco-2) and liver (HepG2) cancers. This study provides new insight into the potential bioactive properties, most importantly the antioxidant activity of *K. uvaria*. In general, different cell lines respond differently to the same compound. So it remains unclear whether similar results would emerge if other cell lines were used alongside the Caco-2, HeLa, and Hep-2 cell lines, hence further studies will be needed to unveil it. The report of Singh and Reddy^17^ mentioned that one of the difficulties encountered during their study was the lack of existing data on the cytotoxicity of *Bulbine* spp. fractions that could. be used to further validate their findings. It is believed that our study has provided more information in addition to the existing data regarding the cytotoxicity of these plants. Phytochemical screening of the fractions may be required to identify the cytotoxic compounds in the fractions before this result can be extrapolated on animal models to study the in vivo effects. This ethnomedicinal knowledge could enhance the efficacy of the already existing or little ethnomedicinal use of these plants.

It is important to mention that further studies are needed to investigate the possible specific targets involved in the cytotoxic pathway of each plant extracts using molecular techniques.
